# Complete Genomes of *Bacillus coagulans* S-lac and *Bacillus subtilis* TO-A JPC, Two Phylogenetically Distinct Probiotics

**DOI:** 10.1371/journal.pone.0156745

**Published:** 2016-06-03

**Authors:** Indu Khatri, Shailza Sharma, T. N. C. Ramya, Srikrishna Subramanian

**Affiliations:** CSIR-Institute of Microbial Technology, Sector 39A, Chandigarh, India; University of Hyderabad, INDIA

## Abstract

Several spore-forming strains of *Bacillus* are marketed as probiotics due to their ability to survive harsh gastrointestinal conditions and confer health benefits to the host. We report the complete genomes of two commercially available probiotics, *Bacillus coagulans* S-lac and *Bacillus subtilis* TO-A JPC, and compare them with the genomes of other *Bacillus* and *Lactobacillus*. The taxonomic position of both organisms was established with a maximum-likelihood tree based on twenty six housekeeping proteins. Analysis of all probiotic strains of *Bacillus* and *Lactobacillus* reveal that the essential sporulation proteins are conserved in all *Bacillus* probiotic strains while they are absent in *Lactobacillus* spp. We identified various antibiotic resistance, stress-related, and adhesion-related domains in these organisms, which likely provide support in exerting probiotic action by enabling adhesion to host epithelial cells and survival during antibiotic treatment and harsh conditions.

## Introduction

Probiotics are increasingly being used as prophylactics for gastrointestinal disorders and as nutritional supplements or novel foods to promote good health. Most commonly used probiotic bacteria are autochthonous, lactic acid bacteria belonging to the genera, *Lactobacillus* and *Bifidobacterium*. Many commercially available probiotics today are also bacterial spore-formers, mostly GRAS strains of the genus, *Bacillus* [1]. While *Bacillus* species were originally believed to be strictly soil bacteria and therefore regarded as allochthonous probiotics, there is now evidence that strains of *Bacillus subtilis* and probably other species are human gut commensals, too [2]. Members of *Bacillus* genus form resistant dormant endospores as a protective mechanism during conditions of nutrient deprivation and environmental stress, which makes them resistant to extreme pH, UV irradiation, high temperatures, and solvents, and allow them to be stored without refrigeration [3]. Consequently, *Bacillus* probiotics are currently of keen interest to the probiotic industry as they can be marketed in the spore form, which has indefinite shelf life [1]. The tough coat of the spores helps these organisms to transit across gastric environmental barriers, and experiments conducted using a murine model have demonstrated that ingested *Bacillus subtilis* spores actually germinate, proliferate and re-sporulate in the gut [4]. Probiotic *Bacillus* species available in the market include *Bacillus subtilis*, *Bacillus cereus*, *Bacillus licheniformis*, *Bacillus pumilus*, *Bacillus clausii* and *Bacillus coagulans* [1]. Other probiotic spore-formers include *Brevibacillus laterosporus* and *Paenibacillus polymyxa* [1].

Various studies have demonstrated the safety, bile and acid tolerance, mucin binding, and immune stimulation ability of probiotic *Bacillus* strains and their clinical efficacy in gastrointestinal disorders [5–11]. The production of antibiotics, bacteriocins and lytic enzymes with antimicrobial activity, the secretion of amylolytic and pectinolytic enzymes that support digestive function in the gut and the production of essential amino acids and vitamins possibly contribute to the probiotic effects of *Bacillus* bacteria [12].

*Bacillus subtilis* has been extensively studied at genetic and physiological levels [5]. *Bacillus subtilis* produces the bacteriocins, subtilin and subtilosin. *Bacillus subtilis* has been demonstrated to improve clinical, microbiologic and immunologic efficacy in acute infectious diarrhea in young children [13]. *Bacillus subtilis* has been shown to suppress traveler’s diarrhea caused by the pathogen *Citrobacter rodentium* in a murine model [14]. In poultry, *Bacillus subtilis* has been shown to suppress pathogenic infections caused by *Salmonella enterica* [15], *Clostridium perfringens* [15], and *Escherichia coli* [16]. An *in vitro* study has shown potential for use against *Helicobacter pylori* [17].

*Bacillus coagulans* [18] is unique in that it shows characteristics of both Bacillaceae and Lactobacillaceae and this together with the phenotypic heterogeneity of this species [18] has made its taxonomic position with respect to these two families difficult [19]. It shares certain characteristics such as the production of lactic acid, lack of cytochrome-c oxidase and inability to reduce nitrate to nitrite with the genus *Lactobacillus*; however, it is catalase positive and forms spores, like other *Bacillus* members and in contrast to *Lactobacillus* members [19–21]. The endospore location is terminal in *Bacillus coagulans* unlike *Bacillus subtilis* and other members of *Bacillus* where it is sub-terminal or central.

*Bacillus coagulans* strain I_4_ produces the plasmid-borne coagulin [22] and lactosporin with anti-microbial activity against pathogenic microorganisms [23]. *Bacillus coagulans* has been demonstrated to significantly improve abdominal pain and bloating associated with irritable bowel syndrome in two double-blind, randomized, placebo-controlled clinical trials [24, 25]. *Bacillus coagulans* has also been shown to be effective in the form of a synbiotic in improving symptoms of irritable bowel syndrome and childhood functional abdominal pain [26, 27]. In a study of 40 Indian women, *Bacillus coagulans*, as an adjunct to antibiotic therapy has been demonstrated to have a positive effect in the treatment of bacterial vaginosis [28]. *Bacillus coagulans* has also been demonstrated to reduce symptoms of *Clostridium difficile*-induced colitis in mice [29, 30]. Additionally, a study measuring the *in vitro* T-cell response of individuals consuming *Bacillus coagulans* suggests that this probiotic might increase immune response to viral infections [31]. A *Bacillus coagulans* strain has also been shown to have a growth promoting, prophylactic effect on broiler chickens [32].

For the current study, we selected two commercially available spore-forming probiotics, *Bacillus coagulans*, and *Bacillus subtilis*. *Bacillus coagulans* is marketed by Sanzyme Ltd., Hyderabad in India with the trade name Sporlac® and labeled “Lactic Acid *Bacillus* (earlier known as *Lactobacillus sporogenes)”*. *Bacillus subtilis* TO-A JPC is a constituent of Vibact®, a probiotic formulation, labeled as containing four bacterial strains—*Bacillus mesentericus TO-A JPC*, *Lactic acid bacillus (Lactobacillus sporogenes)*, *Streptococcus faecalis T-110 JPC* and *Clostridium butyricum TO-A*, and manufactured by Allianz Biosciences Pvt. Ltd., Pondicherry, India licensee of TOA Pharmaceuticals Co. Ltd., Japan. Both *Bacillus coagulans* and *Bacillus subtilis* probiotics are widely available in the Indian market and are popularly prescribed by physicians for use as an adjunct to antibiotic therapy to prevent antibiotic-induced diarrhea and/or other gastrointestinal distress. Considering their wide usage and their limited genome-level characterization, we selected these spore-forming probiotic strains for whole genome sequencing. We have analyzed their genomes and performed comparative analysis at the genomic level with other members of their species as well as with other probiotic strains of *Bacillus* and *Lactobacillus* in order to glean insights into the molecular basis of probiotic function exerted by these microbes.

## Results and Discussion

### Assembly of complete genomes from PacBio long reads

**Fig 1:**
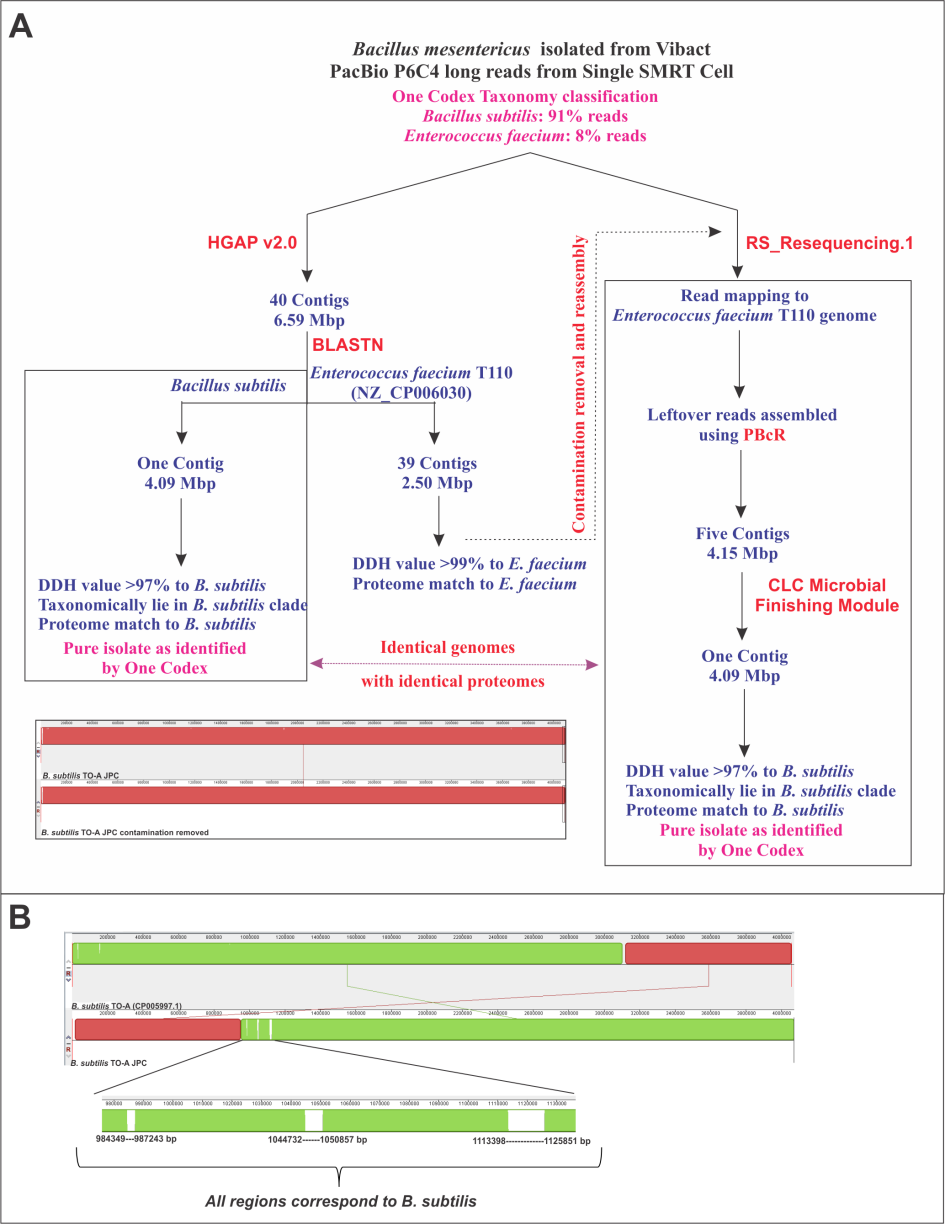
The assembly and contamination check workflow for the *B*. *subtilis* genome.

We assembled *de novo* the complete circular genomes of *Bacillus coagulans* S-lac and *Bacillus subtilis* TO-A JPC, using HGAP v2.0, from PacBio reads obtained using the P6C4 chemistry (**Table 1**). We obtained a single contig of 3.69 Mbp corresponding to a complete and circular chromosome for *Bacillus coagulans* S-lac. PacBio reads for *Bacillus subtilis* TO-A JPC assembled in 40 contigs. We subjected these contigs to BLASTn against the NT database of NCBI. The largest contig of 4.09 Mbp, which assembled as a circular chromosome showed extensive matches to nucleic acid sequences of *Bacillus subtilis* members. The remaining 39 contigs found matches to sequences from *Enterococcus faecium* T110 genome (NZ_CP006030.1) indicating that *Enterococcus faecium* T110 was present as a contaminant. We subjected the long PacBio reads to Onecodex [33] and found that 8% of the reads correspond to *Enterococcus faecium* T110 genome and 91% to *Bacillus subtilis*. No matches to components of Vibact® capsules were obtained. The PacBio reads corresponding to the contaminant were removed by read mapping to *Enterococcus faecium* T110 genome. The assembly performed after removal of *Enterococcus faecium* reads yielded a 4.09 Mbp genome, which was complete, circular and concordant to the largest contig from the earlier assembly. We subjected the Coding Sequence (CDS) regions of the genome to BLASTp against NR database and found that all coding sequences match *Bacillus subtilis* proteins, and none found top scoring matches to any other *Bacillus* spp. Further, the assembled genome of *Bacillus subtilis* TO-A JPC was aligned to the *Bacillus subtilis* TO-A (CP005997.1) genome determined independently by another group using Illumina data, and we found that the two assemblies were concordant (**Fig 1**).

**Table 1:** Genome assembly and annotation statistics of *Bacillus coagulans* S-lac and *Bacillus subtilis* TO-A JPC.

	*Bacillus coagulans* S-lac	*Bacillus subtilis* TO-A JPC
**Sequencing technology**	**PacBio P6C4**	**PacBio P6C4**
**Bio Project Number**	**PRJNA224116**	**PRJNA224116**
**NCBI Accession number**	**CP011939**	**CP011882**
**Genome size (in bp)**	**3,694,837**	**4,090,708**
**GC content (%)**	**46.2**	**43.8**
**CDS**	**4088**	**4231**
**Coding density (%)**	**86**	**89**
**Hypothetical proteins**	**1510**	**1029**
**tRNA**	**83**	**87**
**rRNA operon**	**10**	**10**
**Restriction-Modification systems**	**Type I and III**	**Type II**
Clustered regularly interspaced short palindromic repeats **(CRISPR) systems**	**Type II**	**RNase III**

In *Bacillus coagulans* S-lac the replication origin was identified at 1,091,681–1,092,581 bp and the corresponding *dnaA* gene was located downstream of the replication origin at 1092593–1093942 bp. It shows maximum similarity with *Bacillus coagulans* 36D1 replication origin (ORI95010925, 912 bp) with an E-value of 0.0, 99% identity, and 100% coverage. Similarly, in *Bacillus subtilis* TO-A JPC the replication origin was identified at 952,081–952,992 bp and the corresponding *dnaA* gene was located downstream of the replication origin at 952993–954333 bp. It shows maximum similarity with *Bacillus subtilis subsp*. *natto* BEST195 replication origin (ORI96010027, 912 bp) with an E-value of 0.0, 99% identity, and 100% coverage.

### *In silico* DDH values and whole-genome alignments reveal heterogeneity within strains of *B*. *coagulans*

The *in silico* DNA-DNA-hybridization (DDH) values were calculated using Genome-Genome Distance Calculator (GGDC) 2.0 server [34] by subjecting the complete genome of *Bacillus coagulans* S-lac and *Bacillus subtilis* TO-A JPC to hybridization against all the publically available complete genomes of the genera, *Bacillus* and *Lactobacillus*. With Formula II, which uses the length of high scoring pairs for calculation instead of the genome length, our sequenced strain of *Bacillus coagulans* was closest to *Bacillus coagulans* strain 36D1 with 87% DDH value followed by *Bacillus coagulans* strain 2–6 (with 60% DDH value) suggesting significant genome variations between these strains (**Table 2**). The complete genome of *Bacillus coagulans* S-lac, when aligned to draft probiotic *Bacillus coagulans* GBI-30 (224 contigs) using Mauve Contig Mover [35], aligned as a single block, whereas there were various rearrangements when aligned with *Bacillus coagulans* 36D1 and *Bacillus coagulans* 2–6 complete genomes (**S1 Fig**). The other species of *Bacillus* genus had <40% DDH values suggesting that the *Bacillus coagulans* genome has significantly diverged from the other *Bacillus* spp. A maximum DDH value of ~97% was obtained for *Bacillus subtilis* TO-A JPC when hybridized with *Bacillus subtilis* subsp. *subtilis* str. 168 (**Table 3**). The genome of *Bacillus subtilis* TO-A JPC aligned with *Bacillus subtilis subsp*. *subtilis* str. 168, *Bacillus subtilis subsp*. *subtilis* 6051-HGW and *Bacillus subtilis* QB928 in four blocks, but there were no rearrangements within strains suggesting a high degree of similarity with each other (**S2 Fig**).

**Table 2:** DDH values as calculated by GGDC server with *B*. *coagulans* as query against other *Bacillus* strains as reference. The values are sorted on Formula II.

Query genome	Reference genomes	Formula I	Formula II	Formula III
*B*. *coagulans* S-lac	*B*. *coagulans* 36D1	89.1	87.4	91.5
*B*. *coagulans* S-lac	*B*. *coagulans* P38	86.2	82.6	88.5
*B*. *coagulans* S-lac	*B*. *coagulans* NL01	84.2	81.1	86.6
*B*. *coagulans* S-lac	*B*. *coagulans* GBI30	100	73.2	98.8
*B*. *coagulans* S-lac	*B*. *coagulans* 2–6	75.4	60	74.8
*B*. *coagulans* S-lac	*B*. *cereus* FRI-35	12.7	41.3	13.2
*B*. *coagulans* S-lac	*B*. *anthracis* str. Ames	12.7	40.9	13.1
*B*. *coagulans* S-lac	*B*. *halodurans* C-125	12.7	40.3	13.1
*B*. *coagulans* S-lac	*B*. *cytotoxicus* NVH 391–98	12.8	40	13.2
*B*. *coagulans* S-lac	*B*. *infantis* NRRL B-14911	12.7	39.3	13.2
*B*. *coagulans* S-lac	*B*. *weihenstephanensis* KBAB4	12.8	39.2	13.2
*B*. *coagulans* S-lac	*B*. *thuringiensis* HD-771	12.7	39.1	13.1
*B*. *coagulans* S-lac	*B*. *toyonensis* BCT-7112	12.8	39	13.2
*B*. *coagulans* S-lac	*B*. *pseudofirmus* OF4	12.7	39	13.1
*B*. *coagulans* S-lac	*B*. *thuringiensis* BMB171	12.8	38.8	13.2
*B*. *coagulans* S-lac	*B*. *cellulosilyticus* DSM 2522	12.7	38.6	13.1
*B*. *coagulans* S-lac	*B*. *licheniformis* 9945A	12.7	37.1	13.2
*B*. *coagulans* S-lac	*B*. *clausii* KSM-K16	12.7	36.9	13.1
*B*. *coagulans* S-lac	*B*. *megaterium* DSM 319	12.8	35.1	13.2

**Table 3:** DDH values as calculated by GGDC server with *B*. *subtilis* TO-A JPC as query against other *Bacillus* strains as reference. The values are sorted on Formula II.

Query genome	Reference genome	Formula I	Formula II	Formula III
*B*. *subtilis* TO-A JPC	*B*. *subtilis* subsp. *subtilis* 6051-HGW	97.9	97.4	98.8
*B*. *subtilis* TO-A JPC	*B*. *subtilis* TO-A (CP005997.1)	100	97.2	100
*B*. *subtilis* TO-A JPC	*B*. *subtilis* subsp. *subtilis* str. 168	97.9	97.1	98.7
*B*. *subtilis* TO-A JPC	*B*. *subtilis* QB928	97.5	97	98.5
*B*. *subtilis* TO-A JPC	*B*. *subtilis* PY79	99.3	96.9	100
*B*. *subtilis* TO-A JPC	*B*. *subtilis* subsp. *subtilis* str. BSP1	95.9	90.5	96.9
*B*. *subtilis* TO-A JPC	*B*. *subtilis* BSn5	95.7	90	96.7
*B*. *subtilis* TO-A JPC	*B*. *subtilis* subsp. *natto* BEST195	84.2	86.9	87.6
*B*. *subtilis* TO-A JPC	*B*. *subtilis* subsp. *subtilis* str. BAB-1	94.2	84.9	95
*B*. *subtilis* TO-A JPC	*B*. *subtilis* XF-1	93.5	84.7	94.5
*B*. *subtilis* TO-A JPC	*B*. *subtilis* subsp. subtilis str. RO-NN-1	93.7	83.4	94.4
*B*. *subtilis* TO-A JPC	*B*. sp. JS	90.3	63	88
*B*. *subtilis* TO-A JPC	*B*. *subtilis* subsp. spizizenii TU-B-10	86.2	50.8	81
*B*. *subtilis* TO-A JPC	*B*. *subtilis* subsp. spizizenii str. W23	85.8	49.6	80.2
*B*. *subtilis* TO-A JPC	*B*. *cellulosilyticus* DSM 2522	12.7	41.1	13.1
*B*. *subtilis* TO-A JPC	*B*. *coagulans* 36D1	12.8	38.1	13.2
*B*. *subtilis* TO-A JPC	*B*. *coagulans* 2–6	12.8	37.8	13.2
*B*. *subtilis* TO-A JPC	*B*. *toyonensis* BCT-7112	12.8	36.4	13.2
*B*. *subtilis* TO-A JPC	*B*. *thuringiensis* MC28	12.8	36.2	13.2

### Phylogenetic analysis to reveal taxonomic position of *Bacillus coagulans* and *Bacillus subtilis*

Members of *Bacillus* genus have undergone much re-classification with respect to their taxonomic position at species and genus levels. *Vibrio subtilis* was renamed *Bacillus subtilis* in 1872, and similarly, *Bacillus coagulans* was originally named *Lactobacillus sporogenes*, then reclassified as *Bacillus sporogenes* and subsequently as *Bacillus coagulans* [18].

**Fig 2:**
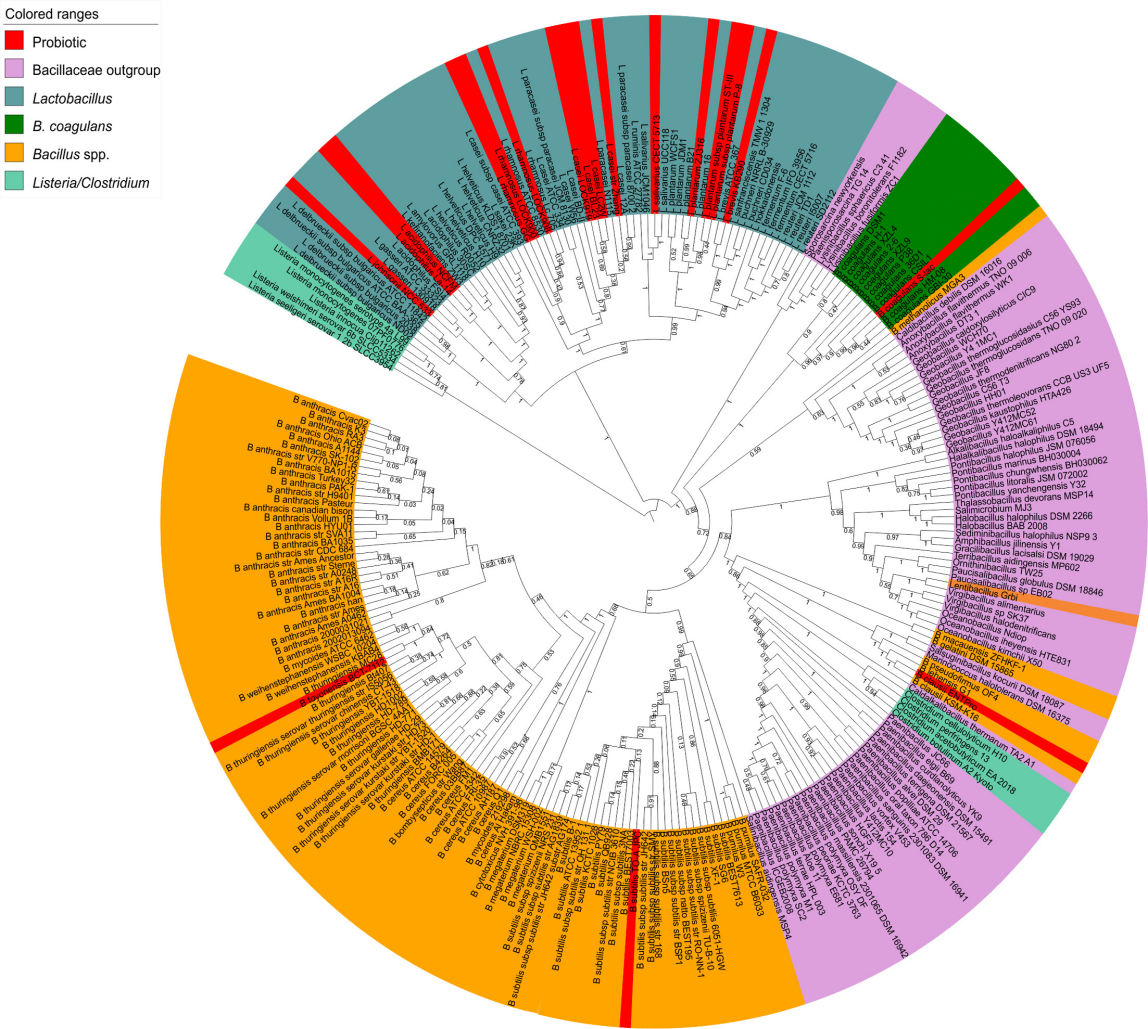
26 Housekeeping ML-based phylogenetic analysis of members of genus *Bacillus*, *Lactobacillus*, *Clostridium* and *Listeria* and other members of family Bacillaceae. The species have been colored according to the genus they belong to: *Bacillus* spp. are colored orange, outgroups of Bacillaceae family are colored dark-pink, outgroup members related to Clostridiaceae and Listeriaceae family are colored dark-blue, *Lactobacillus* spp. are colored blue. The organisms that have been used as probiotics are colored red.

The protein sequences of twenty-six housekeeping genes were retrieved from the members of *Bacillus* spp., *Listeria* spp., *Clostridium* spp., *Lactobacillus* spp. and the members of Bacillaceae family. The concatenated protein sequences were aligned using MUSCLE [36] and the phylogenetic tree was built using RAxML v8.0 [37]. The ML phylogenetic tree is divided into nine clades **(Fig 2)**. *Listeria* spp., as a separate clade, is at the root of the phylogenetic tree. The members of *Lactobacillus* spp. form a separate clade. Four clades comprise members of Bacillaceae family and one clade comprises all the strains of *B*. *coagulans*. The members of *Bacillus* genus are grouped in two clades which are further divided into separate species specific clades such as *Bacillus anthracis*, *Bacillus subtilis*, *Bacillus thuringiensis*, *Bacillus amyloliquefaciens*, *Bacillus cereus* and other species. There is no intermixing of these organisms within clades. In this phylogeny *B*. *coagulans* has formed a separate clade which suggests that this organism is an outgroup *Bacillus* species. *Bacillus subtilis* TO-A JPC groups within the *Bacillus subtilis* clade.

### Core proteome analysis

**Fig 3:**
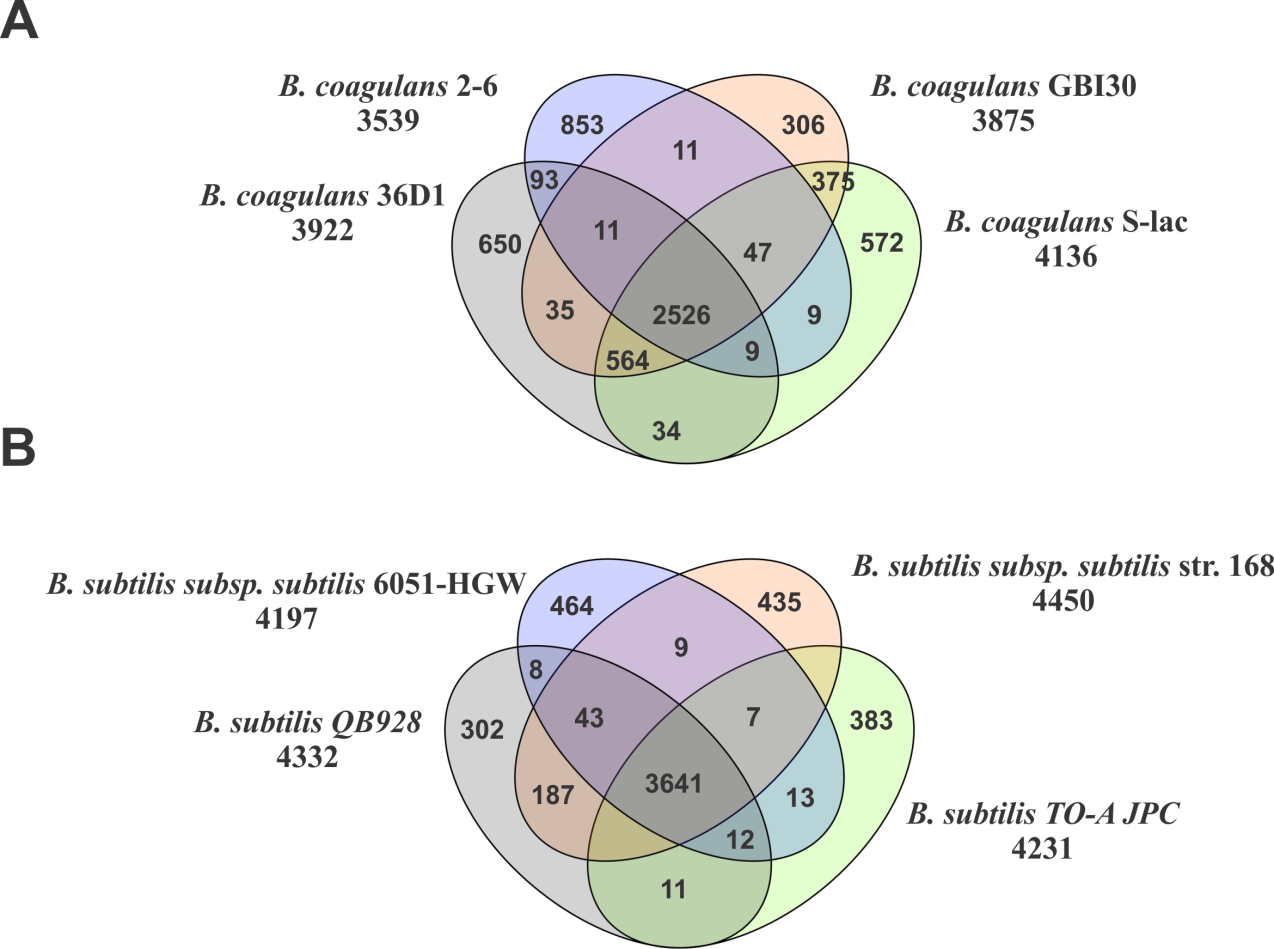
**Core, dispensable and unique sets of proteins a)** Orthologous set of proteins in *Bacillus coagulans* S-lac, *Bacillus coagulans* GBI-30, *Bacillus coagulans* 36D1 and *Bacillus coagulans* 2–6 **b)** Orthologous set of proteins in *Bacillus subtilis* TO-A JPC, *Bacillus subtilis* subsp. *subtilis* str. 168, *Bacillus subtilis* subsp. *subtilis* 6051-HGW and *Bacillus subtilis* QB928.

Orthologous proteins among different strains of *Bacillus coagulans* and *Bacillus subtilis* were obtained by all-versus-all reciprocal BLAST using Proteinortho v2.3 [38] Perl script. The analysis revealed that 2198 proteins formed the core set of proteins among all the members of *Bacillus coagulans* considered here. There were 2787 core proteins sharing orthology across all the members of *Bacillus subtilis*. We plotted the orthologous proteins in orthology chart for the three organisms closest to each of the two probiotic organisms of interest. We compared *Bacillus coagulans* S-lac with *Bacillus coagulans* GBI-30 with which it shares the maximum number of orthologous pairs (3512), with *Bacillus coagulans* 36D1 with which it shares 3090 orthologous proteins and with *Bacillus coagulans* strain 2–6 with which it shares 2526 proteins. All four strains share 2526 orthologs (**Fig 3A**). We compared *Bacillus subtilis* TO-A JPC with *Bacillus subtilis subsp*. *subtilis* str. 168 (with which it shares 3812 proteins), *Bacillus subtilis* subsp. subtilis 6051-HGW (with which it shares 3673 proteins) and *Bacillus subtilis* QB928 (with which it shares 3792 proteins) (**Fig 3B**). These four strains share 3641 orthologs. DDH values and analysis of shared orthologous pairs revealed that *Bacillus coagulans* S-lac is closest to *Bacillus coagulans* GBI-30 genome and *Bacillus subtilis* TO-A JPC is closest to *Bacillus subtilis* subsp. *subtilis* str. 168, both of which are draft genomes.

We also performed the reciprocal all versus all BLAST on all the *Bacillus* and *Lactobacillus* probiotic species to find the conserved proteins among the probiotics of these related genera. The organisms compared are listed in **Table 4**.

**Table 4:** The list of probiotic *Bacillus* and *Lactobacillus* compared.

Organism	Genome Accession Number
*Bacillus clausii* ENTPro	CP012475.1
*Bacillus toyonensis* BCT-7112	CP006863.1
*Bacillus coagulans* GBI-30	JPSK00000000.1
*Bacillus paralicheniformis* BL-09	CP010524.1
*Lactobacillus acidophilus* La-14	CP005926.2
*Lactobacillus acidophilus* NCFM	CP000033.3
*Lactobacillus brevis* KB290	AP012167.1
*Lactobacillus casei* BL23	FM177140.1
*Lactobacillus casei* LC2W	CP002616.1
*Lactobacillus casei* LOCK919	CP005486.1
*Lactobacillus casei* str. Zhang	CP001084.2
*Lactobacillus johnsonii* NCC 533	AE017198.1
*Lactobacillus paracasei* N1115	CP007122.1
*Lactobacillus plantarum* subsp. *plantarum* P-8	CP005942.2
*Lactobacillus plantarum* subsp. *plantarum* ST-III	CP002222.1
*Lactobacillus plantarum* ZJ316	CP004082.1
*Lactobacillus rhamnosus* GG	FM179322.1
*Lactobacillus rhamnosus* LOCK900	CP005484.1
*Lactobacillus rhamnosus* LOCK908	CP005485.1
*Lactobacillus salivarius* CECT 5713	CP002034.1

**Fig 4:**
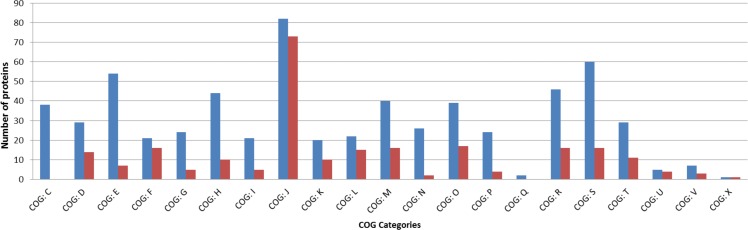
COG categories conserved across *Bacillus* probiotics vs. *Lactobacillus* probiotics. The orthologous proteins conserved across *Bacillus* probiotics and distribution of proteins in different COG categories are plotted in comparison to COG categories conserved across the *Bacillus* and *Lactobacillus* probiotics. X-axis represents the number of proteins and Y-axis represents the COG categories.

We retrieved seven hundred and seven proteins as pairs of orthologs among the *Bacillus* probiotics, and the number of orthologous protein pairs was reduced to two hundred and fifty-seven proteins when we added probiotic members of *Lactobacillus* genus to the analysis. We subjected these orthologous protein pairs to BLASTp against the COG database [39, 40] for identifying the conserved functions among *Bacillus* and *Lactobacillus* probiotics. We found that category C corresponding to energy production and conversion was conserved across the *Bacillus* probiotics but were not present as conserved orthologs among *Bacillus* and *Lactobacillus* probiotics. *Bacillus* probiotics alone had a significant number of orthologous pairs of proteins belonging to the following categories—Amino acid transport and metabolism (COG: E), Coenzyme transport and metabolism (COG: H), Lipid transport and metabolism (COG: I), Inorganic ion transport and metabolism (COG: N) and General function (COG: P) (**Fig 4**).

### Analysis of unique proteome reveals horizontal gene transfers in *Bacillus coagulans*

Apart from the core and dispensable orthologs present across all strains of an organism, the proteome could contain homologs of non-orthologs of the related strains analyzed. Thus, the proteins that were remaining with no orthologous pairs within each other were extracted from the proteomes of *Bacillus coagulans* S-lac (572 proteins) and *Bacillus subtilis* TO-A JPC (383 proteins) and subjected to BLASTp against NR database. Out of the 383 proteins in *Bacillus subtilis* TO-A JPC only six did not find any hit to any sequence in the database and 377 proteins had homology to *Bacillus subtilis* proteins at >90% identity.

**Fig 5:**
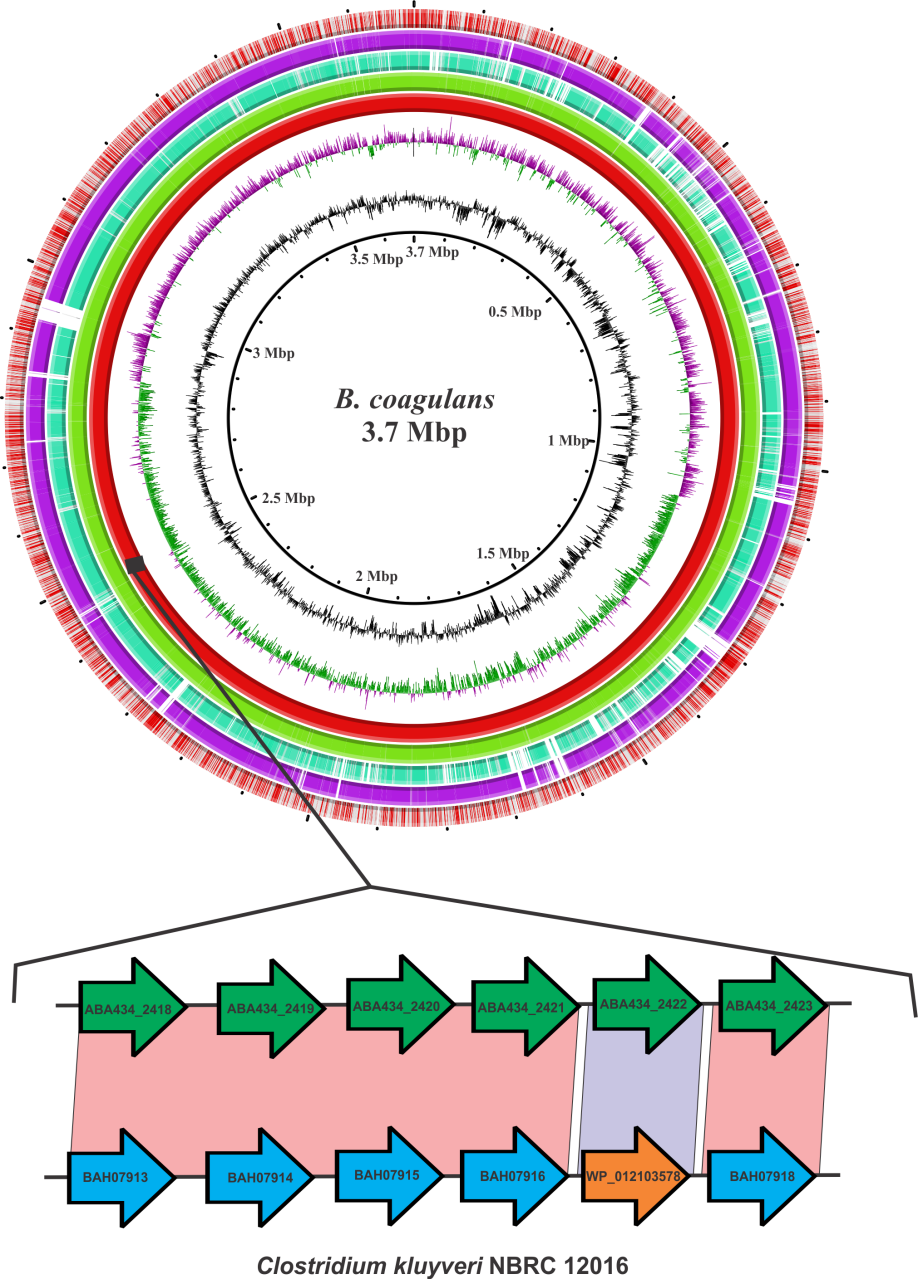
Horizontal gene transfers mapped onto the circular genome for *Bacillus coagulans* S-lac. Circles (from inside to outside) 1 and 2 represent GC content and GC skew, Circle 3 (red colored) is the genome itself; circle 4 (green colored) represent the *Bacillus coagulans* 2–6 genome mapped on *the Bacillus coagulans* S-lac. Similarly, circle 5 (cyan colored) and 6 (magenta colored) are *Bacillus coagulans* GBI-30 and *Bacillus coagulans* 36D1 mapped respectively on *Bacillus coagulans* S-lac. Circle 7 is the adhesion, sporulation and stress-related proteins that have been mapped onto the genome. The genes corresponding to *Clostridium kluyueri* genome represents the RM Type III system which lies on a phage element that is also present in *Bacillus coagulans* strain GBI-30.

Out of the 572 proteins in *Bacillus coagulans* S-lac, 86 proteins did not find a significant match to any sequence in the NR database. These were of very short length (20–40 residues) and were annotated as hypothetical proteins. Apart from these 86 proteins, 31 proteins were fetched from the *Bacillus coagulans* S-lac proteome, of which 12 proteins had a lower percent identity (<90%) to *Bacillus coagulans* proteins and the remaining 19 proteins found top matches to proteins from other *Bacillus* spp. or from other genera of Firmicutes. These 19 proteins were studied for their syntenic regions, if any, to check if they were true horizontal transfers. Most of these proteins were pseudogenes but interestingly, we found a homolog of the *Bacillus coagulans* protein, AB434_2420 in *Clostridium kluyueri* at 100% identity. On investigating the syntenic regions, we found that proteins AB434_2418 to AB434_2423 of *Bacillus coagulans* were 70–99% identical to the proteins from *Clostridium kluyueri*. These syntenic proteins include phage protein, Primase, hypothetical protein, Type III RM system subunits, and ATP-dependent endonucleases, and correspond to proteins WP_012103572, WP_012103574, WP_012103576, WP_012103577, WP_012103578 and WP_012103579 of *Clostridium kluyueri* **(Fig 5)**.

### Conserved set of sporulation proteins in probiotics

Spore-forming probiotics have an advantage over non-spore formers as spores are resistant to harsh environmental conditions, unlike vegetative cells. The mechanism of sporulation is highly sophisticated and a large number of proteins (approximately 600) function in a concerted fashion at various stages [41]. The core set and the complete set of sporulation proteins that are required during the different stages of sporulation in *Bacillus* and *Clostridium* species have been grouped into different categories: 70 proteins in general sporulation genes, 19 in Small Acid-soluble Spore Proteins (SASPs), 18 in spore cortex, 78 in spore coat formation, 17 in spore coat maturation, 28 in spore coat germination, 18 in cell division and DNA replication, 51 in cell wall metabolism, 15 in housekeeping, seven in signaling, 15 in Spo0A phosphorylation, 19 in transcriptional regulation and 25 in transport across spore coat [41]. Certain proteins were identified through transcriptional profiling to have direct or indirect roles in sporulation for which no specific roles were traced and thus are grouped in uncharacterized (209 proteins) and poorly characterized (30 proteins) proteins. Out of a total of 619 proteins, 72 proteins have been mentioned to be functioning as essential sporulation proteins [41].

We extracted all the sporulation proteins from Genbank and subjected them to BLASTp against the proteome of *Bacillus coagulans* strain S-lac. We manually confirmed all the matches obtained and also employed alternate searches using keywords for those queries with no hits. We found that *Bacillus coagulans* S-lac harbored ~56% of these well-characterized sporulation proteins and *Bacillus subtilis* TO-A JPC harbored 81% of these sporulation proteins–the highest among all probiotic bacilli. Members of *Lactobacillus* spp. do not sporulate and so are not expected to contain proteins related to sporulation. It was therefore not surprising that all these sporulation proteins were absent in all members, including the probiotic species, of this genus. All the Bacillus probiotic genomes had minimal sets of sporulation proteins (**Table 5**). The essential set of sporulation proteins [41] were present in all the probiotic genomes studied.

**Table 5:** Distribution of sporulation proteins in probiotic *Bacillus* genomes.

Categories	Total sporulation genes	*Bacillus coagulans* S-lac	*Bacillus clausii* ENTPro	*Bacillus coagulans* GBI-30	*Bacillus toyonensis* BCT-7112	*Bacillus paralicheniformis* BL-09	*Bacillus subtilis* TO-A JPC
Cell wall metabolism	51	27	18	17	20	37	49
Cell division DNA replication	18	13	10	12	15	18	17
General sporulation	70	63	59	58	65	65	66
Housekeeping	15	10	9	5	13	11	12
Small acid-soluble spore protein	19	13	7	10	10	15	17
Signaling	7	5	4	3	5	7	7
spo0A phosphorylation	15	6	8	5	11	5	11
Spore coat	78	23	25	27	37	40	64
Spore coat maturation	17	1	6	1	3	2	17
Spore cortex	18	13	9	7	12	16	16
Spore germination	28	12	13	11	17	21	24
Transcription regulation	19	10	11	12	10	14	19
Transport	25	17	17	9	16	18	23
Poorly characterized	30	13	16	14	14	20	30
Uncharacterized	209	51	86	73	82	102	131

Among general sporulation proteins, the sporulation proteins are further divided into 0-VI stages according to their function and their recruitment. We found that all stage 0 sporulation proteins were present in *Bacillus coagulans* S-lac except Spo0M. Similarly, all stage II sporulation proteins (SpoIIAA, SpoIIAB, SpoIID, SpoIIE, SpoIIGA, SpoIIM, SpoIIp, SpoIIQ, SpoIIR, SigE, SigF, SigG, and SigH) were present, except for SpoIIQ2 in the probiotic genomes *Bacillus coagulans* S-lac and *Bacillus subtilis* TO-A JPC and SpoIIB in *Bacillus coagulans*. The proteins involved in Stage III, IV, V and VI of sporulation were all present in both organisms. The presence and distribution of general sporulation proteins in *Bacillus toyonensis* BCT-7112, *Bacillus clausii* ENTPro, *Bacillus paralicheniformis* BL-09, and *Bacillus coagulans* GBI-30 were observed to be similar to that in *Bacillus coagulans* strain S-lac.

In *Bacillus subtilis* TO-A JPC, all the sporulation kinases, KinA-D, that initiate the sporulation process as a response to different environmental stimuli were present whereas only KinB is present in *Bacillus coagulans* S-lac. Regarding other *Bacillus* probiotic genomes, we found a maximum of eight sporulation kinases present in *Bacillus toyonensis* BCT-7112 and just two kinases in *Bacillus clausii* ENTPro and *Bacillus paralicheniformis* BL-09—KinA and KinB, and KinA and KinC, respectively. The larger number of kinases in *Bacillus toyonensis* suggests its response to a wide range of environmental stimuli as compared to those with a single sporulation kinase. On analyzing the distribution of the sporulation proteins in their respective categories, we found that the proteins belonging to spore coat maturation, poorly characterized and uncharacterized categories were represented by a few proteins from all *Bacillus* probiotics except *Bacillus subtilis* TO-A JPC.

Not surprisingly (because the sporulation query database comprises *Bacillus subtilis* sequences), we observed that most sporulation proteins were identified in *Bacillus subtilis*. We found only 63% of sporulation proteins in *Bacillus paralicheniformis* and 40–44% in *Bacillus coagulans* suggesting that the taxonomic proximity of *Bacillus* members to *Bacillus subtilis* group could account for the increase in the percent of sporulation proteins identified. A similar trend was reported previously; the presence and the number of sporulation genes were found to correlate with taxonomic proximity [41].

### Antimicrobial molecules in *Bacillus* probiotics

Bacteriocins are ribosomally synthesized antimicrobial polypeptides that provide the organism a selective advantage over other strains [22]. Bacteriocins have the significant potency to combat intestinal infections caused by drug-resistant strains of pathogenic bacteria and have been employed against pathogens such as *Listeria monocytogenes*, *Clostridium difficile*, *Helicobacter pylori*, *Gardenerella vaginalis*, *Staphylococcus aureus* and *Streptococcus pneumoniae* [42]. Bacteriocins are stable, have low toxicity, have narrow- or broad-spectrum activity, and are amenable to bioengineering [42]. Bacteriocins in Gram-positive bacteria are usually membrane-permeabilizing cationic peptides with <60 residues [43]. *Bacillus subtilis* is known to produce the bacteriocins, subtilin, and subtilosin, and *Bacillus coagulans* has been documented to produce plasmid-borne coagulin [22] and lactosporin [23].

The BAGEL3 database [44], a comprehensive database of bacteriocins, classifies bacteriocins in three classes—modified, unmodified and >10 kDa bacteriocins, and these further into various sub-classes according to their properties. In the BAGEL3 database, seven modified bacteriocins of *Bacillus subtilis* have been grouped into three sub-classes lanthipeptide A, sactipeptide and glycocin, and three bacteriocins are listed in the unmodified class. The *Bacillus coagulans* bacteriocin (coagulin) is classified under the unmodified class of bacteriocins. The BAGEL3 database lists a lanthipeptide in *Bacillus paralicheniformis*, but no bacteriocins for *Bacillus clausii* and *Bacillus toyonensis* (**S1 File**).

We identified four CDS coding for subtilosin (ABU16_0601, ABU16_0603, ABU16_0605, and ABU16_0608) in the *Bacillus subtilis* TO-A JPC genome by RAST annotation. However, when we investigated, these genes, we found that only one CDS codes for subtilosin; others were not subtilosin homologs. Genes from ABU16_0601 to ABU16_0608 belong to a *sbo*-*alb* gene cluster, which is required for synthesis of subtilosin [45]. Subjecting *Bacillus subtilis* TO-A JPC genome to BAGEL3, we were able to identify two sactipeptides. One of these sactipeptides corresponds to subtilosinA and the other to sporulation killing factor skfA. SubtilosinA is 93% identical to a bacteriocin reported earlier in *Bacillus subtilis* subsp. *subtilis* strain 168 (A24125), and sporulation killing factor skfA is 98% identical to that reported in *Bacillus subtilis* (WP_019257315) (**Table 6**). With BAGEL3, we predicted the bacteriocins in other strains and subspecies of *Bacillus subtilis* but all were homologous to the previously known bacteriocins. Thus, we did not predict any new sub-class of bacteriocins in any of the *Bacillus subtilis* strains.

**Table 6:** The bacteriocins identified in *Bacillus* probiotics with their matches in NR database.

Organism	Bacteriocin Sub-class	Accession Number	% Identity	Matching organisms
***B*. *coagulans* S-lac**	Head to tail cyclized peptide	WP_017550351	100%	*Bacillus coagulans*
	Head to tail cyclized peptide	**No match in NR database**		
***B*. *coagulans* GBI-30**	Head to tail cyclized peptide	WP_017550351	100%	*Bacillus coagulans*
***B*. *coagulans* 2–6**	bacteriocin >10kd	WP_013858635	100%	*Bacillus coagulans*
	Head to tail cyclized peptide	WP_017550351	100%	*Bacillus coagulans*
***B*. *coagulans* CSIL1**	Sactipeptides	**No match in NR database**		
***B*. *coagulans* 36D1**	Head to tail cyclized peptide	WP_035190623	100%	*Bacillus coagulans*
***B*. *coagulans* HM-08**	Head to tail cyclized peptide	**No match in NR database**		
	Head to tail cyclized peptide	**No match in NR database**		
***B*. *coagulans* H1**	Sactipeptides	**No match in NR database**		
***B*. *clausii* ENTPro**	Lanthipeptide class I in contig 20	WP_048311475	75%	*Anaerobacillus macyae*
***B*. *paralicheniformis* BL-09**	Lanthipeptide class II	**No match in NR database**		
	Head to tail cyclized peptide	WP_020452200	100%	*Bacillus*
	Lasso peptide	WP_026580277	100%	*Bacillus*
***B*. *toyoensis* BCT-7112**	Lasso peptide	WP_000260275	95%	*Bacillus cereus*
	bacteriocinII	WP_000190134	100%	*Bacillus cereus*
	LAPs	BAL17023.1	97%	*Bacillus cereus*
***B*. *subtilis* TO-A JPC**	Sactipeptides	A24125	93%	*Bacillus subtilis*
	Sactipeptides	WP_019257315	98%	*Bacillus subtilis*
***B*. *subtilis* subsp. *subtilis* str. 168**	Sactipeptides in ABQK010000021	A24125	93%	*Bacillus subtilis*
	Glyocin in ABQK010000051	WP_009967544	100%	*Bacillus subtilis*
	Sactipeptides in ABQK010000015	WP_019257315	98%	*Bacillus subtilis*
***B*. *subtilis* subsp. s*ubtilis* 6051 HGW**	Sactipeptides	A24125	93%	*Bacillus subtilis*
	Glyocin	WP_009967544	100%	*Bacillus subtilis*
	Sactipeptides	WP_019257315	98%	*Bacillus subtilis*
***B*. *subtilis* QB928**	Sactipeptides	A24125	93%	*Bacillus subtilis*
	Glyocin	WP_009967544	100%	*Bacillus subtilis*
	Sactipeptides	WP_019257315	98%	*Bacillus subtilis*
***B*. *subtilis* 3NA**	Sactipeptides	A24125	93%	*Bacillus subtilis*
	Glyocin	WP_009967544	100%	*Bacillus subtilis*
	Sactipeptides	WP_019257315	98%	*Bacillus subtilis*

Using BAGEL3, we found several bacteriocins previously unknown in *Bacillus coagulans*. We predicted two head to tail cyclized peptides in *Bacillus coagulans* S-lac and *Bacillus coagulans* HM08, of which one is a novel bacteriocin with no matches in the NR database. We predicted a single head to tail cyclized peptide corresponding to known circularin in *Bacillus coagulans* strain GBI-30. We predicted *Bacillus coagulans* CSIL1 to have a single sactipeptide with no matches in the NR database. Thus, we have found one head to tail cyclized peptide in *Bacillus coagulans* S-lac and *Bacillus coagulans* HM08 and one sactipeptide in *Bacillus coagulans* CSIL1, which are novel bacteriocins with no previous instances reported in the NR database. We could not identify coagulin; this bacteriocin is plasmid borne [22], and the presence of plasmids in these *Bacillus coagulans* strains has not been previously reported.

Using BAGEL3, we were also able to predict bacteriocins previously unknown in other probiotic *Bacillus* species. The lanthipeptide in *Bacillus clausii* belongs to gallidermin class with maximum 75% identity to the antibiotic protein of *Anaerobacillus macyae* (WP_048311475). Using BAGEL3 analysis for the genome of the probiotic, *Bacillus paralicheniformis* BL-09, one novel bacteriocin was identified belonging to lanthipeptide Class II.

Using BAGEL3 analysis for the genome of the probiotic, *Bacillus toyonensis* BCT-7112, we revealed the presence of three bacteriocin clusters with four bacteriocins belonging to three subclasses. One bacteriocin in sub-class lasso peptide was 95% identical to metallo-phosphoesterase of *Bacillus cereus* (WP_000260275), and one in subclass LAPs Pfam domain (PF10169) was 97% identical to hypothetical protein in *Bacillus cereus* (BAL17023.1). There were two bacteriocins identified in a single cluster within a subclass of unmodified bacteriocins, which find matches to peptidase M23 of *Bacillus cereus* (WP_000190134) and bacteriocin biosynthesis protein of *Bacillus cereus* (WP_000615928), suggesting a putative transfer between these two groups.

The *Bacillus subtilis* genome has been investigated intensely for its potential in industrial use since the beginning of genomics era, so it is not surprising that no new bacteriocin was identified. The novel bacteriocins we have identified in *Bacillus coagulans*, *Bacillus clausii*, and *Bacillus paralicheniformis* can be further explored for their use as antimicrobial agents.

### Antibiotic resistant proteins

Antibiotic resistance is a common and natural phenomenon in Gram-positive bacteria. It is achieved by acquisition of genes horizontally through plasmids or transposons, or foreign DNA recombination into the chromosome, or mutations at different chromosomal loci [46]. Several beneficial Gram-positive bacteria such as *Lactobacillus*, *Bifidiobacterium*, (a common inhabitant of human gut microbiota) and representatives of *Bacillus* have been used in the probiotic industry [47]. Regulators prefer probiotic strains to carry as few acquired resistance genes as possible, so that they are not a source for donating these genes to other bacteria including pathogens. In the Indian context of prescription of probiotics as an adjunt to antibiotic therapy, antibiotic-resistant probiotics would help in restoring the antibiotic-sensitive normal gut microbiota and prevent antibiotic-associated diarrhea and/or gastrointestinal distress. However, this practice obviously constitutes a safety concern due to the possibility of transfer of antibiotic resistance to gut flora (which can then serve as a reservoir for antibiotic resistance) and to pathogenic bacteria in the intestine via plasmids or transposons [47, 48].

We did not find any evidence for the presence of plasmids in *Bacillus subtilis* TO-A JPC and *Bacillus coagulans* S-lac, thus the gain and transfer of any plasmid-borne antibiotic-resistance genes are not obvious at this stage. Nevertheless, we searched for the presence of antibiotic resistance genes and related efflux pumps in the chromosomes. The proteomes of probiotic *Bacillus* spp. and *Lactobacillus* spp. were subjected to hmmscan against Resfams database [49] and we identified 93 Resfams domains imparting antibiotic resistance to different antibiotics to these organisms. The Resfams domains were further grouped on the basis of their ‘mechanism classification’ and their presence-absence variations were plotted (**S2 File**).

Most of these domains were all present across the probiotics with the maximum number of antibiotic resistance domains present in *B*. *toyoensis* BCT-7112 followed by *B*. *paralicheniformis* BL-09, *B*. *clausii* ENTPro and members of *B*. *subtilis*. *B*. *coagulans* and *Lactobacillus* have a similar domain distribution. All probiotics contain a large number of ABC transporters with the maximum present in *B*. *clausii* ENTPro and *B*. *toyoensis* BCT-7112 followed by *L*. *casei*, *L*. *rhamnosus*, and *B*. *paralicheniformis* BL-09. ABC transporters were larger in number in *Lactobacillus* spp. as compared to *B*. *coagulans* but MFS transporters were more abundant in *B*. *coagulans* as compared to *Lactobacillus* spp. Antibiotic inactivation mechanism was absent in members of both *B*. *coagulans* and *Lactobacillus* spp. but was present in members of *B*. *subtilis*; the antibiotic inactivation mechanism was also absent in other *Bacillus* probiotics.

Domains imparting tetracycline resistance are divided based on four different mechanisms as MFS efflux, tetracycline inactivation, tetracycline resistance and tetracycline ribosome protection in Resfams [49]. Here, in these probiotic proteomes only the domains related to MSF efflux and tetracycline ribosome protection were identified whereas the MSF efflux domains were completely absent in *B*. *clausii* ENTPro and members of *Lactobacillus* spp. and *B*. *coagulans*. Tetracycline ribosome protection function was present across all the probiotics.

β-lactamases impart resistance against a broad spectrum of antibiotics and are divided into five sub-classes: Classes A-D and Unclassified in Resfams database [49] of which Class C could not be identified in the *Bacillus* and *Lactobacillus* probiotics. We found that Class A and Class D β-lactamases were absent in the members of *B*. *coagulans* and *Lactobacillus* spp. but were present in other *Bacillus* probiotics and members of *B*. *subtilis*. Class B β-lactamase domains in all the *Bacillus* probiotics were approximately double in number as compared to *Lactobacillus* probiotics. We would like to add a cautionary note that earlier studies have shown that an organism may display intrinsic resistance to certain antibiotics that are not gene borne [47, 48]. While we have attempted to correlate the genome-level presence and absence of domains imparting antibiotic resistance to their probable phenotypes, we have not conducted any phenotypic studies to corroborate these analyses and/or check for intrinsic resistance.

### Adhesion proteins

Probiotic organisms adhere to the mucous membrane and epithelial cells of the intestine to exert their probiotic effect. The specialized mucin and fibrinogen binding proteins in probiotics bind to the digestive tract and reduce pathogenic adherence [50]. We looked in Pfam for the keywords, adhesion, colonization, mucin-binding and fibrinogen-binding, and further searched for and retrieved each entry with a role in adhesion. In all, 289 Pfam domains selected from 776 matches to keywords were searched across the complete proteomes of all probiotics.

We observed the three mucin-binding protein domains, PF06458, PF04650, and PF13350, only in the *Lactobacillus* probiotics but not in any of the *Bacillus* genomes, save for the PF13350 domain that was present in the proteome of *Bacillus toyonensis* BCT-7112 (**S3 File**). Domain PF00746 known as the Gram-positive anchor is present only in *Bacillus clausii* ENTPro, *Bacillus subtilis* strains and *Lactobacillus* strains. We found the domains present in collagen-binding surface proteins (PF05737 and PF05738) only in *Bacillus clausii* ENTPro, *Bacillus toyonensis* BCT-7112, *Bacillus paralicheniformis* BL-09, certain *Bacillus subtilis* strains and *Lactobacillus* strains but not in *Bacillus subtilis* TO-A JPC, *Bacillus coagulans* strains S-lac and GBI-30. Of Laminin domains I and II, which are glycoproteins, we could only identify the Laminin domain II in *Bacillus coagulans* but found both the domains in *Bacillus clausii* and domain I in *Bacillus paralicheniformis*. The fibronectin-binding protein A N-terminus (FbpA) domain (PF05833) that consists of an N-terminal region of prokaryotic fibronectin binding protein is found in a single copy in all these organisms and likely facilitates attachment of these bacteria to host fibronectin. We observed that the domain Fibronectin-binding protein (FBP), N-terminal (PF07299), which is predicted to play a role in virulence in *Listeria*, was absent in *Bacillus coagulans* and *Bacillus subtilis* probiotic genomes. We identified the domain N_methyl_2 (PF13544) that appears as type IV prokaryotic filamentous adhesins or pilins in three copies in *Bacillus coagulans*, in two copies in *Lactobacillus* probiotics, and in a single copy in *Bacillus subtilis*, *Bacillus clausii*, and *Bacillus paralicheniformis*, indicative of its potential importance in adhesion of *Bacillus coagulans*. We only found the Cell wall binding repeat two family (PF04122) present in adhesins and amidase enhancers in *Bacillus subtilis*, *Bacillus paralicheniformis*, and *Bacillus toyonensis* and not in *Bacillus coagulans*, *Bacillus clausii*, and *Lactobacillus* probiotics. We observed a similar type of domain distribution of most of the domains involved in adherence and attachment of the bacterial cells to the eukaryotic cells across all the organisms compared.

### Stress-responsive proteins

Probiotic organisms, while passing through the gut, face different types of environmental conditions and stress. Spore-former probiotics are ingested in the form of spores that can tolerate much of the environmental stress during its passage through the gut. The spores can germinate into vegetative cells that also have to withstand harsh environmental conditions such as acidic, osmotic, alkaline and oxidative stresses. We extracted 33 stress-resistant and stress related domains from the Pfam database using keyword ‘stress’ and ‘stress-resistance’. We then searched for these 33 stress-resistant and stress related Pfam domains in all the probiotic genomes. We found that 20 of these 33 domains were present in all probiotic organisms (**S4 File**).

The basic stimulus-response coupling by two-component regulatory systems in organisms helps them to sense and respond to the changes in different environmental conditions [51]. We found that the Pfam family HisKA (PF00512) implicated in a wide range of stimuli such as nutrients, cellular redox state, changes in osmolarity, quorum signals, antibiotics, temperature, chemo-attractants and pH was present in eight copies in *Bacillus coagulans*, twenty-one in *Bacillus clausii* ENTPro, thirty-nine in *Bacillus toyonensis* BCT-7112, eighteen in *Bacillus paralicheniformis* BL-09, sixteen in *Bacillus subtilis*, and approximately eight in *Lactobacillus* probiotics (similar to *Bacillus coagulans* and less than other *Bacillus* probiotics).

Further, we studied molecular chaperone proteins like HSP70, HSP90, and DnaJ that provide resistance to heat shock and oxidative stress across all the probiotic genomes. We identified four proteins with HSP70 (PF00012) domain functioning as a molecular chaperone in both *Bacillus coagulans* S-lac and *Bacillus subtilis* TO-A JPC. Also, we identified another molecular chaperone molecule HSP90 represented by the PF00183 Pfam domain in the chaperone protein HtpG in both these organisms. A single copy of the HtpG protein is present in *Bacillus coagulans* S-lac and two copies in *Bacillus subtilis* TO-A JPC. We found another chaperone protein DnaJ in a single copy in both probiotic genomes *Bacillus coagulans* S-lac and *Bacillus subtilis* TO-A JPC, and identified three proteins corresponding to universal stress proteins (PF00582) that provide resistance against environmental stress in *Bacillus coagulans* S-lac and two proteins in *Bacillus subtilis* TO-A JPC. Further, we found that the ankyrin repeats (Ank_2; PF12796 and Ank_4; PF13637) that play a significant role in protein folding and are very common in eukaryotes were absent from all of the *Bacillus* and *Lactobacillus* genomes studied but were present in *Bacillus clausii* ENTPro, *Bacillus toyonensis* BCT-7112 and *Lactobacillus plantarum* ZJ316.

To survive in habitats of varying ionic concentrations, organisms often harbor genes that make them tolerant to divalent metal ions such as cadmium, zinc, and cobalt, and to changes in ionic concentration. We found three domains of each metal ion resistance (PF01545) and varying ionic concentration (PF00122) in *Bacillus coagulans* S-lac and *Bacillus subtilis* TO-A JPC. In *Lactobacillus* genomes, we observed that the domains imparting resistance to change in ionic concentration were in ten copies, which suggest that there could be more resistance to ionic concentration variation in *Lactobacillus* as compared to *Bacillus* probiotics. Heavy metal resistance could be mediated by transport or detoxification of proteins. We found four proteins possibly mediating heavy metal detoxification in *Bacillus coagulans* S-lac and *Bacillus subtilis* TO-A JPC, but the single protein representing PF00403 domain in *Lactobacillus* represents the more efficient heavy metal efflux system in *Bacillus* probiotics. Also, we found two proteins with reductase domains (PF03960) that detoxify arsenate, arsenite, and antimonite in *Bacillus coagulans* and *Lactobacillus* genomes and three such domains in *Bacillus clausii* ENTPro, *Bacillus toyonensis* BCT-7112 and *Bacillus subtilis* genomes.

All aerobic organisms contain certain oxygen scavenging enzymes as the oxidative stress created by O_2_^-^ and H_2_O_2_ can cause cell damage [52]. These proteins belong to AhpC/TSA family (PF00578). We identified Thiol peroxidase, Glutathione peroxidase, copper metallochaperone, Thiol:disulfide oxidoreductase and Alkyl hydroperoxide reductase proteins in all the *Bacillus* probiotics and Thiol peroxidase, Glutathione peroxidase and Thiol:disulfide oxidoreductase and Alkyl hydroperoxide reductase proteins only in *Lactobacillus* probiotics. We found the domain PF00578 present in six proteins in *Bacillus coagulans*, ten proteins in *Bacillus subtilis* and *Bacillus clausii*, eleven proteins in *Bacillus paralicheniformis* BL-09, and nine proteins in *Bacillus toyonensis* BCT-7112. In contrast, we predicted just three domains in *Lactobacillus* probiotics. The distribution of this thiol-dependent antioxidant domain suggests higher oxidative stress resistance in *Bacillus* probiotics as compared to *Lactobacillus* probiotics.

We identified Bile acid sodium symporter that provides the organism resistance to bile salt from the RAST annotation in both *Bacillus coagulans* S-lac (AB434_3605) and *Bacillus subtilis* TO-A JOC (ABU16_1155). Also, we found two genes coding for choloylglycine hydrolase and potentially aiding in bile salt resistance in *Bacillus subtilis* TO-A JPC (ABU16_0835 and ABU16_0836) and *Bacillus coagulans* S-lac (AB434_1322 and AB434_1323).

## Materials and Methods

### Isolation and purification of genomic DNA

*Bacillus coagulans* spore suspension drug Sporlac® (labelled as”*Lactic Acid Bacillus (earlier known as Lactobacillus sporogenes”*), manufactured by Sanzyme Ltd. (Batch No. DSP-14073; Mfd. 06/2014 and Exp 11/2015) and *Bacillus subtilis* (labelled as *Bacillus mesentericus*) from the spore suspension drug Vibact® manufactured by TOA Pharmaceuticals Co., Ltd.” (Batch No. AA4F1; Mfd. 04/2014 and Exp 09/2015) were procured from a local drugstore. The bacteria were cultured, and genomic DNA was isolated. Vibact® powder was suspended in 0.9% saline, serially diluted, and plated on nutrient agar plates. The plates were incubated at 30°C for 48 hours. Sporlac® powder was suspended in 0.9% saline and heated at 65°C for 15 minutes in a water bath followed by homogenization for 5 minutes in a bead beater. The plating was done on Glucose Yeast agar plates at 40°C for 48 hours. DNA isolation was performed using the ZR Fungal/Bacterial DNA miniprep kit (Zymogen) as per instructions in its user manual. The ratio of OD at 260/280 nm was >1.8 as observed by NanoDropND-1000 spectrophotometer.

### Genome Sequencing

Both the genomes were sequenced using PacBio P6C4 chemistry. SMRTbell libraries were created using the ‘Procedure and Checklist–20 kb template preparation using BluePippin^TM^ Size selection system protocol (http://www.pacb.com/wp-content/uploads/2015/09/Procedure-Checklist-20-kb-Template-Preparation-Using-BluePippin-Size-Selection.pdf). DNA samples were sheared and concentrated using AMPure magnetic beads and treated by ExoVII to remove single stranded ends. Blunt ligation reactions were prepared, and SMRTbell templates were purified using AMPure magnetic beads. BluePippin^TM^ size selection was performed to retain longer reads for sequencing. The size-selected SMRTbell templates were annealed, and polymerase was added for sequencing. Single SMRT cells for each library were run on the PacBio RS II system using P6C4 chemistry and an 180-minute data collection mode at the Genome Quebec Centre, McGill University, Canada.

### Genome Assembly and Annotation

The long PacBio reads were assembled *de novo* using HGAP v2.0 in SMRT Portal. Functional annotation was carried out by RAST (Rapid Annotation using Subsystem Technology) [53, 54], tRNA was predicted by tRNAscan-SE 1.23 [55] and rRNA genes by RNAmmer 1.2 [56]. The reads were mapped to plasmid database (downloaded from NCBI on 15-07-2015) for confirming the presence of any plasmids in the probiotic genomes. The *Bacillus coagulans* S-lac genome assembled as a single chromosome whereas *Bacillus subtilis* TO-A JPC was assembled in 40 contigs where one contig was 4.09 Mbp and the remaining 39 add up to 2 Mbp. These 40 contigs were subjected to BLASTn against the NT database of NCBI and the largest contig matched *Bacillus* spp. and the remaining 39 to *Enterococcus faecium* strain T110 (NZ_CP006030.1) [57]. Further, to crosscheck the accurate resolution of the *Bacillus* genome, vis-à-vis the contigs from *Enterococcus faecium*, the reads were mapped to the *Enterococcus faecium* T110 genome and the remaining reads were assembled using the Celera Assembler [58] and reconciled using CLC Microbial Finishing module (www.clcbio.com) and mapped back to the complete 4.09 Mbp contig obtained previously. The assembled genomes were then subjected to BLASTn against oriC sequences available at DoriC database [59].

### Taxonomic Classification

The complete genomes of *Bacillus* and *Lactobacillus* genus were downloaded from Genbank and were annotated using RAST annotation server. The protein sequences corresponding to 26 housekeeping genes (CTP synthase, DNA primase, DNA-directed RNA polymerase beta subunit, LSU ribosomal protein L11p, LSU ribosomal protein L13p, LSU ribosomal protein L16p, LSU ribosomal protein L20p, LSU ribosomal protein L27p, LSU ribosomal protein L3p, LSU ribosomal protein L4p, LSU ribosomal protein L5p, LSU ribosomal protein L6p, LSU ribosomal protein L7/L12, Phosphoglycerate kinase, Ribosome recycling factor, SSU ribosomal protein S10p, SSU ribosomal protein S11p, SSU ribosomal protein S13p, SSU ribosomal protein S2p, SSU ribosomal protein S3p, SSU ribosomal protein S5p, SSU ribosomal protein S9p, tmRNA-binding protein SmpB, transcription termination protein NusA, Translation elongation factor Ts, Translation initiation factor 3) [60] were extracted from all the members of *Bacillus* spp. and *Lactobacillus* spp. Also, other members of phylum Firmicutes in Bacillaceae family *viz*. *Alkalibacillus*, *Amphibacillus*, *Anoxybacillus*, *Cladalkalibacillus*, *Cladibacillus*, *Geobacillus*, *Halalkalibacillus*, *Halobacillus*, *Lentibacillus*, *Lysinibacillus*, *Oceanobacillus*, *Ornithinbacillus*, *Paenibacillus*, *Paucisalibacillus*, *Pontibacillus*, *Salinibacillus*, *Salsuginibacillus*, *Sediminibacillus*, *Terribacillus*, *Thalasobacillus*, *Virgibacillus* and *Clostridium* from family Clostridiaceae and *Listeria* from family Listeriaceae were also downloaded as outgroup species and were annotated using RAST server. The protein sequences corresponding to 26 housekeeping genes were extracted from the outgroup organisms. The protein sequences from all the organisms were concatenated and were aligned using MUSCLE [61] and phylogenetic inferences were drawn by Maximum likelihood [ML] approach based on the Whelan And Goldman (WAG) model (Bootstrap: 100) in RAxML v8 [37]. Gamma evolutionary model was used to model evolutionary rate differences among sites.

### Comparative Genomics

We compared *Bacillus coagulans* strain S-lac and *Bacillus subtilis* TO-A JPC with all probiotic *Bacillus* and *Lactobacillus* genomes viz. *Bacillus clausii* ENTPro, *Bacillus toyonensis* BCT-7112, *Bacillus coagulans* GBI-30, *Bacillus paralicheniformis* BL-09, *Lactobacillus acidophilus* La-14, *Lactobacillus acidophilus* NCFM, *Lactobacillus brevis* KB290, *Lactobacillus casei* BL23, *Lactobacillus casei* LC2W, *Lactobacillus casei* LOCK919, *Lactobacillus casei* str. Zhang, *Lactobacillus johnsonii* NCC 533, *Lactobacillus paracasei* N1115, *Lactobacillus plantarum* subsp. *plantarum* P-8, *Lactobacillus plantarum* subsp. *plantarum* ST-III, *Lactobacillus plantarum* ZJ316, *Lactobacillus rhamnosus* GG, *Lactobacillus rhamnosus* LOCK900, *Lactobacillus rhamnosus* LOCK908 and *Lactobacillus salivarius* CECT 5713. Along with the genomes of probiotic *Bacillus* and *Lactobacillus*, the genomes we sequenced were also compared with other members of their respective species. Antibiotic resistance proteins were identified by subjecting the proteomes of the probiotic *Bacillus* and *Lactobacillus* organisms to hmmscan against the Resfams database [49]. All probiotic genomes of *Bacillus* spp. and *Lactobacillus* spp. were scanned using Hidden Markov Model (HMM) [62, 63] for the presence of specific domains involved in acid tolerance, adhesion, heavy metal resistance, bile resistance, oxidative and universal stress resistance. BAGEL3 [44] was used for predicting short ORFs corresponding to bacteriocins and their neighbors. CRISPR elements were identified using CRISPRs finder [64]. Ortholog groups of proteins were searched using Proteinortho v2.3 [38] PERL script.

## Conclusions

The complete circular genomes of the 3.7 Mbp *Bacillus coagulans* strain S-lac and 4.09 Mbp *Bacillus subtilis* TO-A JPC have been assembled using HGAPv2.0 pipeline from PacBio P6C4 chemistry at ~200x coverage. We were able to successfully resolve the circular *Bacillus subtilis* TO-A JPC genome from the long PacBio reads despite the presence of contaminating reads of *Enterococcus faecium*. Considering the cost of sequencing, we believe that our bioinformatics method of exclusion of contaminating sequences will be a valuable strategy not only for assembly of whole genomes from inadvertently contaminated cultures but also for the assembly of whole genomes from sequences of closely associated and difficult-to-isolate organisms such as the algal and fungal components of lichens.

As mentioned earlier, owing to similarities to both Bacillaceae and Lactobacillaceae, the taxonomic positioning of *Bacillus coagulans* has been difficult [18, 19]. With the whole genomes in hand, we were able to build an ML tree based on 26 housekeeping protein sequences, that clearly places *Bacillus coagulans* in a separate clade as an outgroup *Bacillus* species but close to other members of the Bacillaceae family. Our ML tree thus supports its current taxonomic classification in the Bacillaceae family while also demonstrating its unique position with respect to other *Bacillus* species. Interestingly, we also found that Type III RM system in the *Bacillus coagulans* S-lac has possibly transferred horizontally from *Clostridium kluyveri* and was present exclusively in the *Bacillus coagulans* strains, S-lac and GBI-30.

We chose to study *Bacillus coagulans* and *Bacillus subtilis* TO-A JPC because these two strains and another probiotic strain, *Bacillus clausii* are widely available in Indian markets and popularly prescribed for use as an adjunct with antibiotic therapy to prevent antibiotic-induced diarrhea and gastrointestinal disorders. We were therefore interested in analyzing the genomes to unravel biological features that might help understand their mechanism of probiotic action. Our bioinformatics mining approaches helped identify in both these probiotic genomes, sporulation proteins as well as various other proteins that might play a role in probiotic function such as adhesins, which might aid in attachment to host tissues, bacteriocins, that might aid in selectively killing pathogenic and/or commensal microbes in the gut and thus bring about renewed homeostasis, and stress-responsive proteins, which might enable survival in the harsh conditions of the host gastrointestinal tract.

One of our most important findings of this study is the identification of two head to tail cyclized bacteriocins in *Bacillus coagulans* S-lac, of which one is a novel bacteriocin as it did not match any sequence in the NR database. We also identified two cyclized peptides in *Bacillus subtilis* TO-A JPC along with a CDS coding for the bacteriocin, subtilosin, which is well-characterized. The novel bacteriocins we have identified in *Bacillus coagulans*, *Bacillus clausii*, and *Bacillus paralicheniformis* can be further explored for their use as antimicrobial agents in future studies. Biochemical characterization of the antimicrobial activity of these novel bacteriocins would likely provide further clues to the mechanism of probiotic action of these organisms.

Analysis of the genomes of *Bacillus coagulans* S-lac and *Bacillus subtilis* TO-A JPC indicated the presence of genes required for sporulation, as expected. While both these organisms have the minimal set of sporulation genes, and all *Bacillus* probiotics have all the essential sporulation genes, it is interesting that there is variation in the number of the sporulation kinases, KinA-D, that initiate the sporulation process in different probiotics, suggesting variable response to different environmental stimuli.

Analysis of antibiotic resistance genes indicated that larger numbers of MFS and other efflux transporters are present in *Bacillus* probiotics as compared to *Lactobacillus* probiotics. Tetracycline MFS efflux and class A and class D domains of β-lactamases were found to be absent from *B*. *coagulans* and *Lactobacillus* spp. whereas they were present across other *Bacillus* probiotics.

We identified proteins that likely mediate adhesion of these two probiotics. The Gram-positive anchor domain and the Cell wall binding repeat two family domain were found in *Bacillus subtilis* TO-A JPC, and the Laminin domain II in *Bacillus coagulans*. Type IV prokaryotic filamentous adhesins or pilins were identified in *Bacillus coagulans* as well as *Bacillus subtilis*.

While the identification of the genes of interest, by itself, is insufficient to explain the presumed probiotic action, we believe our study paves the way for future experimental characterization of these gene products and a deeper understanding into mechanism of probiotic action of these important microbes.

## Supporting Information

S1 Fig Mauve contig mover alignment of *B*. *coagulans* S-lac as reference with other strains of *B*. *coagulans* with maximum DDH values. A) Mauve contig mover alignment performed for *B*. *coagulans* S-lac as reference with *B*. *coagulans* 36D1 (complete) B) Mauve contig mover alignment performed for *B*. *coagulans* S-lac as reference with *B*. *coagulans* 2–6 (complete) C) Mauve contig mover alignment performed for *B*. *coagulans* S-lac as reference with *B*. *coagulans* GBI-30 (draft)
(TIF)

S2 Fig Mauve contig mover alignment of *B*. *subtilis* TO-A JPC as reference with other strains of *B*. *subtilis* with maximum DDH values. A) Mauve contig mover alignment performed for *B*. *subtilis* TO-A JPC as reference with *B*. *subtilis* subsp. *subtilis* str. 168 (draft) B) Mauve contig mover alignment performed for *B*. *subtilis* TO-A JPC as reference with *B*. *subtilis* subsp. *subtilis* str. 6051-HGW (complete) C) Mauve contig mover alignment performed for *B*. *subtilis* TO-A JPC as reference with *B*. *subtilis* QB928 (complete).
(TIF)

S1 File The list of bacteriocins and the putative regions coding for bacteriocins in the genome of all the strains of *Bacillus coagulans*, *Bacillus subtilis* and probiotic strains of *Bacillus* and *Lactobacillus* genus. (XLSX)

S2 File Presence-absence variation of antibiotic resistance proteins in all the strains of *Bacillus coagulans*, *Bacillus subtilis* and probiotic strains of *Bacillus* and *Lactobacillus* genus. (XLSX)

S3 File Presence-absence variation of adhesion proteins in all the strains of *Bacillus coagulans*, *Bacillus subtilis* and probiotic strains of *Bacillus* and *Lactobacillus* genus. (XLSX)

S4 File Presence-absence variation of stress-responsive proteins in all the strains of *Bacillus coagulans*, *Bacillus subtilis* and probiotic strains of *Bacillus* and *Lactobacillus* genus. (XLSX)

## Abbreviations

ANI: Average Nucleotide Identity
BAGEL: BActeriocin GEnome mining tool
BLAST: Basic Local Alignment Search Tool
COG: Cluster of Orthologous Groups
CRISPRs: Clustered regularly interspaced short palindromic repeats
CDS: Coding DNA Sequences
DDH: DNA-DNA hybridization
GGDC: Genome-Genome Distance Calculator
HGAP: Hierarchical Genome-Assembly Process
HMM: Hidden Markov Model
GRAS: Generally Regarded As Safe
ML: Maximum Likelihood
NCBI: National Centre for Biotechnology Information
NR: Non-Redundant
PERL: Practical Extraction and Reporting Language
RAST: Rapid Annotation using Subsystem Technology
WAG: Whelan and Goldman
