# Supplementary figures and images for: Complete Genomes of Bacillus coagulans S-lac and Bacillus subtilis TO-A JPC, Two Phylogenetically Distinct Probiotics
Source: PLoS One. 2016 Jun 3;11(6):e0156745. doi: 10.1371/journal.pone.0156745 (PMC4892684; doi:10.1371/journal.pone.0156745)

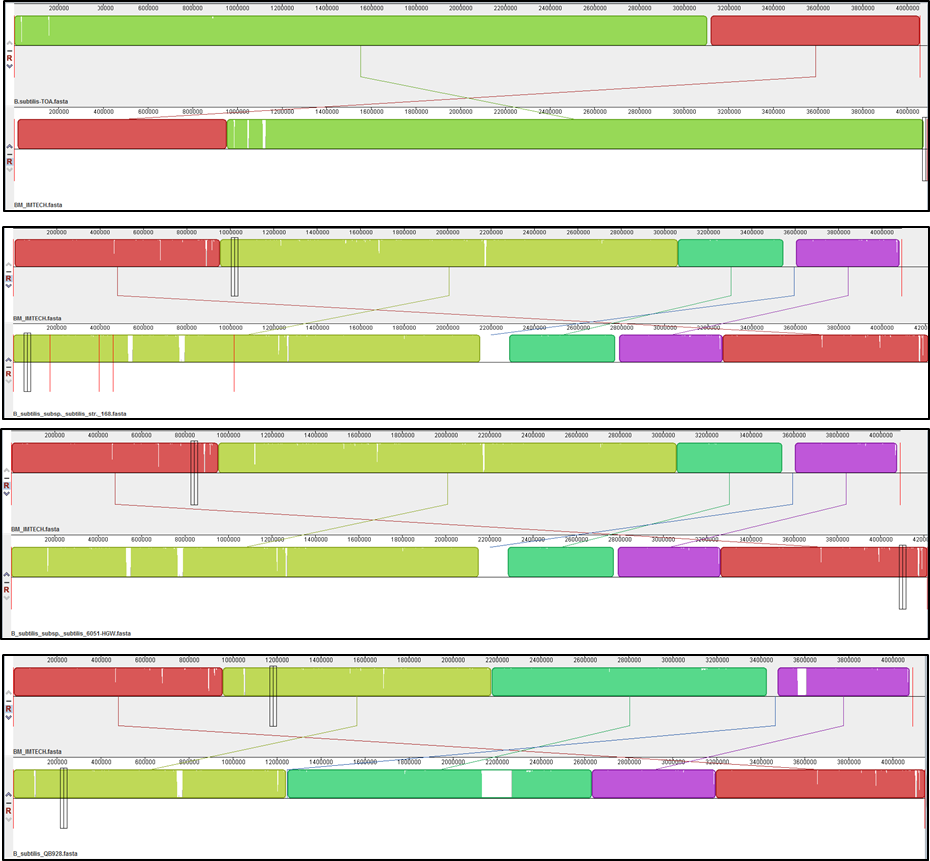

Supplement: S1 Fig — A) Mauve contig mover alignment performed for B. coagulans S-lac as reference with B. coagulans 36D1 (complete) B) Mauve contig mover alignment performed for B. coagulans S-lac as reference with B. coagulans 2–6 (complete) C) Mauve contig mover alignment performed for B. coagulans S-lac as reference with B. coagulans GBI-30 (draft) (TIF) [file pone.0156745.s001.tif]

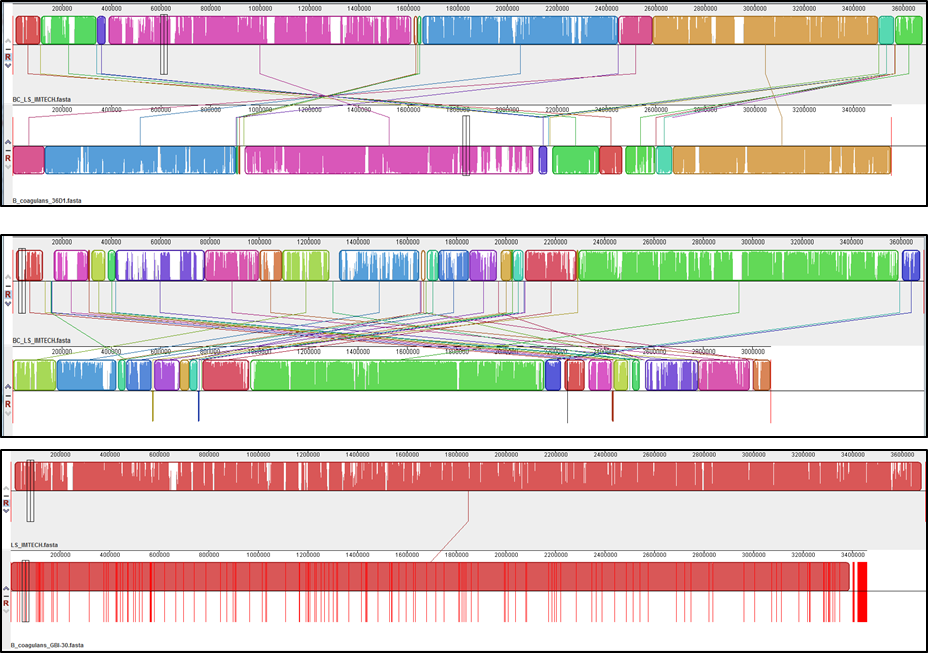

Supplement: S2 Fig — A) Mauve contig mover alignment performed for B. subtilis TO-A JPC as reference with B. subtilis subsp. subtilis str. 168 (draft) B) Mauve contig mover alignment performed for B. subtilis TO-A JPC as reference with B. subtilis subsp. subtilis str. 6051-HGW (complete) C) Mauve contig mover alignment performed for B. subtilis TO-A JPC as reference with B. subtilis QB928 (complete). (TIF) [file pone.0156745.s002.tif]
